# Complete Genome Sequence of the Opitutaceae Bacterium Strain TAV5, a Potential Facultative Methylotroph of the Wood-Feeding Termite Reticulitermes flavipes

**DOI:** 10.1128/genomeA.00060-15

**Published:** 2015-03-05

**Authors:** Malini Kotak, Jantiya Isanapong, Lynne Goodwin, David Bruce, Amy Chen, Cliff S. Han, Marcel Huntemann, Natalia Ivanova, Miriam L. Land, Matt Nolan, Amrita Pati, Tanja Woyke, Jorge L. M. Rodrigues

**Affiliations:** a Department of Biology, University of Texas, Arlington, Texas, USA; b King Mongkut’s University of Technology North Bangkok, Bangkok, Thailand; c Los Alamos National Laboratory, Los Alamos, New Mexico, USA; d DOE Joint Genome Institute, Walnut Creek, California, USA; e Oak Ridge National Laboratory, Oak Ridge, Tennessee, USA; f Department of Land, Water and Air Resources, University of California-Davis, Davis, California, USA

## Abstract

The Opitutaceae bacterium strain TAV5, a member of the phylum *Verrucomicrobia*, was isolated from the wood-feeding termite hindgut. We report here its complete genome sequence, which contains a chromosome and a plasmid of 7,317,842 bp and 99,831 bp, respectively. The genomic analysis reveals genes for methylotrophy, lignocellulose degradation, and ammonia and sulfate assimilation.

## GENOME ANNOUNCEMENT

The ability to grow on single-carbon compounds other than CO_2_ is a distinctive feature of methylotrophs. Among this group, members of the *Verrucomicrobia* have been identified as the first phylum outside of the *Proteobacteria* to be characterized as methanotrophs (1). The genomes of three species isolated from geographically distant but geothermally similar environments were previously sequenced and studied (2–4). Here, we report the complete genome of the Opitutaceae bacterium strain TAV5, a mesophilic verrucomicrobium isolated from the hindgut of the wood-feeding termite Reticulitermes flavipes (5), containing genes associated with methylotrophic competency.

The genomic DNA of TAV5 was isolated using a cetyltrimethylammonium bromide method, and the genome was sequenced using a combination of Illumina HiSeq 2000 and 454 FLX Titanium systems. The individual reads were assembled with the Newbler assembler (Roche). Prodigal (6) was used to identify the genes, and manual curation was done using the Joint Genome Institute (JGI) GenePRIMP pipeline (7). The translated protein-coding genes (coding sequences [CDSs]) were used to search the National Center for Biotechnology Information (NCBI) nonredundant, UniProt, TIGRFam, Pfam, PRIAM, KEGG, COG, and InterPro databases, and a product description for each protein was predicted. Noncoding genes and miscellaneous features were predicted using tRNAscan-SE (8), RNAmmer (9), Rfam (10), TMHMM (6), and signalP (11).

The TAV5 genome is composed of a chromosome that is 7,317,842 bp long and contains 6,000 genes, with a G+C content of 63.45%, and a plasmid that is 99,831 bp long and contains 96 genes, with a G+C content of 54.44%. Together, they contain 5,950 CDSs, 48 tRNA genes, and 1 complete rRNA operon. The number of CDSs with a predicted function is 4,086 (67.03% of the total), the number of KEGG orthologs is 1,912 (31.36%), and the number of Cluster of Orthologous Group classifications is 2,984 (48.95%). An analysis of the genome revealed the presence of enzymes for formate metabolism, such as formate dehydrogenase, a formate transporter, and enzymes of the serine pathway, like serine hydroxymethyltransferase, glycerate kinase, and phosphoenolpyruvate carboxykinase.

The TAV5 genome contains a number of glycoside hydrolases (GH) involved in the degradation of cellulose (GH 5 and 9) and hemicellulose (GH 8, 10, 26, 28, and 53), as observed for the TAV1 and TAV2 genomes (12, 13). The genome has genes for the enzymes 3-carboxymuconate cyclase (EC 5.5.1.5) and 4-carboxymuconolactone decarboxylase (EC 4.1.1.44), which are involved in the degradation of protocatechuate that is derived from lignin (14), as well as genes coding for dioxygenases and dienelactone hydrolase (EC 3.1.1.45), known for ring cleavage of aromatic compounds. These enzymes structurally modify lignin, improving the accessibility of polysaccharides to glycoside hydrolases and increasing the efficiency of degradation (15). The TAV5 genome contains the *cbb*_3_-type cytochrome oxidase gene, implying the role of TAV5 in oxygen removal. Furthermore, genes for ammonia and sulfate assimilation, the urea cycle, and nitrogen fixation were observed. The availability of this genome will allow the understanding of metabolic and physiological properties carried out by members of the phylum *Verrucomicrobia* present in the termite hindgut.

### Nucleotide sequence accession numbers.

The complete genome sequence of the Opitutaceae bacterium TAV5 was deposited in GenBank under the accession numbers CP007053.1 (chromosome) and CP007054.1 (plasmid).
